# Analysis of isokinetic muscle function and postural control in individuals with intermittent claudication

**DOI:** 10.1590/bjpt-rbf.2014.0134

**Published:** 2016-01-19

**Authors:** Morgan Lanzarin, Patricia Parizoto, Gilmar M. Santos

**Affiliations:** 1 Centro de Ciências da Saúde e Esportes, Universidade do Estado de Santa Catarina (UDESC), Florianópolis, SC, Brasil

**Keywords:** intermittent claudication, postural control, muscle strength, risk of falls, rehabilitation

## Abstract

**BACKGROUND:**

Intermittent claudication (IC) is a debilitating condition that mostly affects elderly people. IC is manifested by a decrease in ambulatory function. Individuals with IC present with motor and sensory nerve dysfunction in the lower extremities, which may lead to deficits in balance.

**OBJECTIVE:**

This study aimed to measure postural control and isokinetic muscle function in individuals with intermittent claudication.

**METHOD:**

The study included 32 participants of both genders, 16 IC participants (mean age: 64 years, SD=6) and 16 healthy controls (mean age: 67 years, SD=5), which were allocated into two groups: intermittent claudication group (ICG) and control group (CG). Postural control was assessed using the displacement and velocity of the center of pressure (COP) during the sensory organization test (SOT) and the motor control test (MCT). Muscle function of the flexor and extensor muscles of the knee and ankle was measured by an isokinetic dynamometer. Independent t tests were used to calculate the between-group differences.

**RESULTS:**

The ICG presented greater displacement (*p* =0.027) and speed (*p* =0.033) of the COP in the anteroposterior direction (COPap) during the MCT, as well as longer latency (*p* =0.004). There were no between-group differences during the SOT. The ICG showed decreased muscle strength and power in the plantar flexors compared to the CG.

**CONCLUSION:**

Subjects with IC have lower values of strength and muscle power of plantiflexores, as well as changes in postural control in dynamic conditions. These individuals may be more vulnerable to falls than healthy subjects.

## Introduction

Falling is a serious problem among the elderly. About a third of the people over the age of 65 years experiences at least one fall per year in developed countries^1^. Ten to twenty percent of these falls cause serious injury and the need for hospitalization^2^. A recent economic health analysis revealed that falls in the elderly represent a significant economic burden for society^3^.

The etiology of falls is considered multifactorial, involving extrinsic (environmental) and intrinsic factors^4^. Among the intrinsic factors are the declines in postural control^5^, muscle strength^5^^,^ 6 and deficits in gait^5^^,^ 7. Costello and Edelstein^8^ stated that the identification of individuals with functional decline of the lower limbs, especially those with impaired balance, might be important when identifying individuals with a higher risk of falling.

A condition typically known to cause detriment in the function of the lower limbs is intermittent claudication (IC). IC is caused by peripheral arterial obstructive problems, which reduces blood flow in the arterial veins and is characterized by pain in the lower limbs and reduced walking ability^9^. In addition, individuals with IC may have a lower functional capacity and decreased muscle strength^10^. Due to the association between circulatory failure and nerve motor dysfunction of the lower extremities^11^, it is hypothesized that individuals with IC may be susceptible to balance disorders and to a higher risk of falls.

The correlation between IC and the risk of falls has been explored in the literature, but the results are still inconclusive and controversial. Gardner and Montgomery^12^ demonstrated that patients with IC presented with balance disorders by demonstrating less time spent in a one-legged stance and a higher prevalence of falls compared to individuals without IC. Additionally, Mockford et al.^13^ using computerized dynamic posturography (CDP) showed that IC individuals had higher body sway compared to healthy individuals.

However, Arseven et al.^14^ in a prospective study of 86 subjects with IC, found no association between IC decreased postural control and the risk of falls. Moreover, to date we have not been able to find studies that verified the displacement and the average speed of the center of pressure (COP) in the population of IC, parameters traditionally used in the analysis of balance and that are associated with the risk of falls^15^.

Since current studies on the topic "risk of falls and intermittent claudication in the elderly population" did not show consistent and satisfactory results, this study aims to determine whether individuals with IC are susceptible to falls through the measurement of postural control and isokinetic muscle function. 

## Method

### Study design and Ethical Aspects

This is an observational, cross-sectional study, with a comparative base (type case and control). This study was approved by the Research Ethics Committee of the Universidade do Estado de Santa Catarina (UDESC), Florianópolis, SC, Brazil, under the number 274951, and registered in the Brazil platform under the number 06677313.0.0000.0118.

### Subjects

The study included 32 subjects from both sexes: 16 with IC (average age 64 years, SD=6; average weight 76 Kg, SD=11; average height 1.66 meters, SD=0.06) and 16 healthy participants (average age 67 years, SD=5; average weight 73 Kg, SD=5; average height 1.68 meters, SD=0.11).The participants were matched by age, sex and body mass and allocated in two groups: a group with IC (ICG) and a control group (CG). The sample size was calculated based on the study by Câmara et al.^16^, considering 80% of statistical power and 0.05 of significance level. The Gpower3 software (found in web site http://www.gpower.hhu.de) was used for the sample calculation.

The ICG subjects were selected by convenience and recruited at the angiology clinic of the Regional Hospital São José (HRSJ), Santa Catarina, Brazil, using the following inclusion criteria: clinical diagnosis of peripheral occlusive arterial disease (POAD), aged between 60 and 75 years and with IC during the 6 minute walking test. The clinical diagnosis was made by a physician from the angiology unit of the HRSJ using Doppler ultrasound or CT angiography. For the CG, healthy participants with no history of heart disease or peripheral vascular disease previously evaluated by one of the researchers and recruited from the seniors study (NETI) from UDESC, were included.

Asymptomatic individuals with severe POAD and pain at rest, ischemic ulcers or gangrene; clinically unstable (e.g. acute angina, arrhythmia, decompensated congestive heart failure) were excluded from the ICG; and amputees with neurological and orthopedic problems who were unable to perform the tests were excluded from both groups. All study participants were informed about the procedures and signed an informed consent form.

### Procedures

The procedures were conducted in two phases. Phase 1 consisted of the application of an evaluation form which contained subject identification, anthropometric data and history of falls, the International Physical Activity Questionnaire (IPAQ)^17^ and assessment of the 6-minute walking test^18^. This phase was performed by researcher "A". After this phase, subjects remained at rest for a period of 30 minutes for muscle recovery following the walking test. In phase 2 of the study, data from postural control and muscle strength and power output were collected. The order of testing was randomized by a lottery to eliminate potential bias. This phase was performed by researcher "B".

### Six-minute walk test

The 6-minute walk test (6MWT) was used to assess whether subjects could walk the total distance (TD) or the distance that the initial claudication (DIC) occurred. The test was performed according to the American Thoracic Society standards^18^, requiring a digital timer (VOLLO - VL-233), two cones, a sphygmomanometer (PALM HT-1500 NISSEI) and a pulse oximeter (Rossmax SB100). The test consisted of walking a route of 30 meters with turns, delimited by two cones, for a period of 6 minutes.

The subjects were instructed to walk as many laps as they could at their normal speed and to inform the evaluator of any sudden onset of pain. At the onset of pain, the distance was measured (DIC) without interrupting the test. Each individual performed the 6-MWT twice, and the average of the TD and DIC were saved for analysis. The subjects should sit at rest in a chair, located near the starting position, for 10 minutes before the second test starts^18^.

### Postural control

Postural control was measured by computerized dynamic posturography (CDP), using the Smart Equitest^(r)^ Neurocom (NeuroCom System Version 8.3.0., 2010 NeuroCom International Inc^(r)^, Clackamas, OR). This comprises a standing platform with dual force plates that can be rotated to tip the patient forward and backward (termed as sway-referenced support), or in some cases the force plates can be translated to move the patient toward either an anterior or a posterior direction. The patient's feet are centered on the force plates and then face a brightly colored visual surround that is capable of moving relative to the patient (termed as sway-referenced surround). The CDP included a static equilibrium test (Sensory Organization test-SOT) and a dynamic balance test (Motor Control Test-MCT), both with high sensitivity and specificity for detecting abnormalities of balance^19^.

The SOT evaluated the individual's ability to use different postural control systems (i.e. somatosensory, vestibular and visual) in order to keep "in balance" during sensory conflict conditions. The sensory conflicts were produced by visual surroundings or support platform in response to the anterior posterior sway of the patient^20^.

The SOT consisted of six conditions, each with duration of 20 seconds and three repetitions. The test conditions were as follows: (1) eyes open, fixed surface and visual surrounding; (2) eyes closed and fixed surface; (3) eyes open, fixed surface and sway referenced visual surrounding; (4) eyes open, sway referenced surface and fixed visual surrounding; (5) eyes closed and sway referenced surface; and (6) eyes open, sway referenced surface and visual surrounding.

The MCT evaluated the postural responses of the individuals according to the platform translations. Translation sequences were applied in small, medium and large amplitudes in an anterior - posterior direction in order to generate automatic postural responses of each individual. The test was repeated three times at each amplitude and the offset distance and exposure times were set at 5, 10 and 15 cm/s and 250, 300 and 400 ms for small, medium and large amplitudes, respectively^20^.

Through the MCT, it was possible to measure the latency, which was defined as the time (ms) between the beginning of platform translation and the onset of a motor response by the subject. The motor response was defined as a sudden change in the COP position. The data were recorded from 0.5 seconds before translation until 2 seconds after the task, at an acquisition rate of 100 Hz. The system used four algorithms to calculate the latency time and to identify the quality factor which demonstrated how the four algorithms showed the same result^20^.

To perform the CDP, the subjects were informed about the procedure and, with the use of a harness to prevent a fall, were positioned as follows: in the standing position, barefoot on two force platforms and arms by the side. The distance that the feet were apart was standardized by the height of each individual, according to the manufacturer's instructions^20^.

### Muscular strength & power output

To evaluate muscle strength and power output, the isokinetic dynamometer Biodex System 4 (tm) Pro (Biodex Medical Systems, Shirley, NY, USA) was used. The isokinetic evaluation was conducted on the lower limbs, specifically, the knees and ankles. The peak torque, which is the highest peak torque output throughout the range of motion, and muscle power, which is the speed at which the muscles are able to generate work, were measured^21^.

For the evaluation of the knee joint, each subject remained seated, attached to the chair of the dynamometer by stabilization straps with knees flexed at 90°. The ankle muscles were evaluated with the patient seated, attached to the chair with one knee flexed at 30° and foot secured to the platform "foot plate", according to the manufacturer's instructions^22^. The mechanical axis of the dynamometer was centralized with the physiological axis of each joint. The subjects were familiarized with the equipment performing three replications for each test position. It was observed 90 second rest periods for muscle recovery before the beginning of the evaluation.

The angular speeds and the number of repetitions of the tests were determined based on previous studies^16^^,^ 23. The speed of 60°/s was adopted, with five replications, to determine the peak torque, and the speed of 180°/s was adopted, with 10 repetitions, to measure muscle power for both knee flexors and extensors muscles and the ankle dorsiflexors & plantar flexors muscles. Both tests were conducted in a concentric-concentric reciprocal basis. All participants received verbal encouragement from one of the trained researchers in order to encourage maximum force production.

### Data reduction

The data from the CPD were obtained by the Neurocom Balance System Manager software and later treated in MATLAB (*version 8.0, Math Works, Inc* .) to calculate the total range and average speed of the COP.

The amplitude of the COP was calculated from the distance between the maximum and minimum displacement of the COP in the anteroposterior (ACOPap) and medial-lateral (ACOPml) directions. The average speed of the COP (AS) was calculated from the COP displacement divided by the total time of the trial in the anteroposterior (ASap) and medial-lateral (ASml) directions^24^.

Data regarding the peak torque and muscle power were generated by the Biodex advantage software (V.4X) and normalized by the body mass of the participants. There has beenevidence^25^ that body mass influences the magnitude of the parameters provided by the isokinetic test. Therefore, it is necessary to standardize the torque and power values by body mass to allow comparisons between individuals.

### Statistical analysis

The data obtained from the evaluation form were analyzed using descriptive statistics. The homogeneity of the baseline characteristics between groups, such as age, mass, height and body mass index (BMI) was analyzed by an independent t test.

The dependent variables in this study were the COP displacement amplitudes (ACOPap and ACOPml), the average speeds of COP (ASap and ASml), latency time, peak torque and muscle power. First, these variables were analyzed using descriptive statistics, and normality was investigated using the Shapiro-Wilk test. Since the data showed a Gaussian distribution, the t test for independent samples was used to detect the differences between groups. The significance level was set at 0.05. The Statistical Package for Social Sciences (SPSS) 20.0 for Windows was used to perform the analyses.

## Results

Anthropometric and clinical characteristics of the subjects in the study are presented in Table 1. Of note was the significant difference (*p* =0.001) between the 6MWT, which decreased in the IC group (362.2 m, DP=110 m) relative to the healthy group (547.9 m DP=47 m).

**Table 1:** - Anthropometric and some clinical characteristics in subjects with intermittent claudication & normals.

	CI (n=16)	Controls (n=16)	P
Age	64 (6.2)	67.1 (4.9)	0.1
Mass (Kg)	76.3 (11.7)	73.7 (4.9)	0.5
Height (m)	1.66 (0.06)	1.68 (0.11)	0.4
BMI (%)	27.66 (3.7)	25.72 (3)	0.1
Men (%)	14 (87.5%)	12 (75%)	-
Right Dominant (%)	16 (100%)	14 (87.5%)	-
Affected leg Both Left	14 (87.5%) 2 (12.5%)	- -	- -
History of Falling			
	0	0	
6-minute walk test (6MWT)			
DTC - 6 min (m)	362 m. SD=110	547 m. SD=47	0.001*
DCI (m)	261 m. SD=229	-	-
Physical activity level (IPAQ)			
Sedentary	8 (50%)	6 (37.5%)	-
Moderately Active	8 (50%)	5 (31.25%)	-
Active	0	5 (31.25%)	-
Risk factors			
Smokers (%)	14 (87.5%)	10 (62.5%)	-
Smoking load (pack/year)	41. SD=19	29. SD=9	-
Hypertension (%)	16 (100%)	5 (31.25%)	-
Diabetes mellitus (%)	9 (56.25%)	2 (12.5%)	-
Heart disease (%)	8 (50%)	0	-
Stroke (%)	1 (6.25%)	0	-

*=p=0.05.

**Figure 1:**
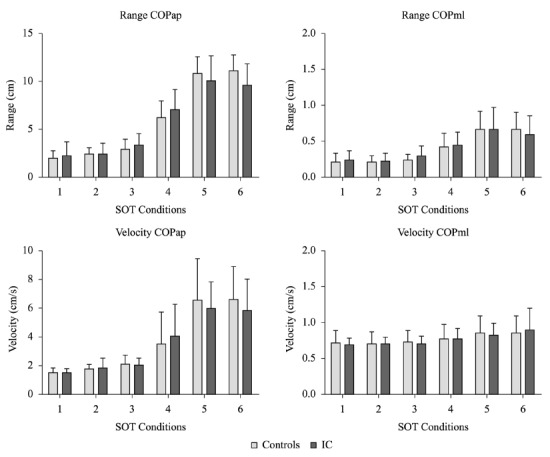
- Sensory Organization Test (SOT) - Range and Velocity of Center of Pressure (COP) in the anteroposterior and mediolateral directions. SOT conditions: (1) eyes open, fixed surface and visual surrounding; (2) eyes closed and fixed surface; (3) eyes open, fixed surface and sway referenced visual surrounding; (4) eyes open, sway referenced surface and fixed visual surrounding; (5) eyes closed and sway referenced surface; and (6) eyes open, sway referenced surface and visual surrounding.

**Figure 2:**
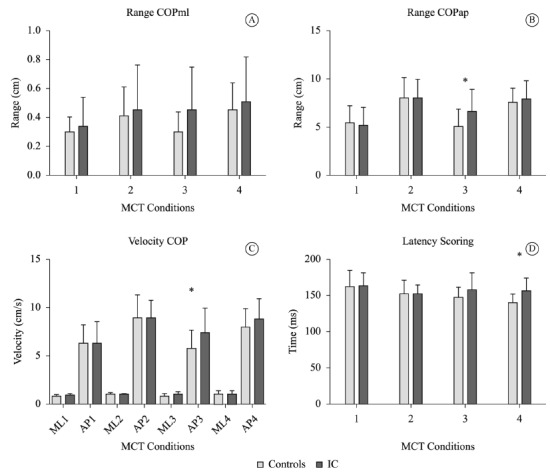
- Control Motor Test - Range and Velocity of Center of Pressure (COP). (A) COP range in the mediolateral direction. (B) COP range in the anteroposterior direction. (C) Average velocity of the COP (ML = mediolateral; AP = anteroposterior). (D) Latency Time. Conditions of Motor Control Test (MCT): 1 = medium platform translation forward; 2 = large platform translation forward; 3 = medium platform translation backward and forward; 4 = large platform translation backward and forward. * = p≤0.05.

The SOTs showed no evidence of statistically significant differences between groups in the amplitude and average speed of the COP for the six test conditions, as shown in Figure 1. During the MCT, the two translations (anterior and posterior) were evaluated at two intensities (medium and large). There were significant differences in mean posterior translation condition with medium intensity at ACOPap (p=0.027) and at ASap (p=0.033). Additionally, there were significant differences in latency time, it was higher in the ICG (156 ms, SD=17) compared to the CG (140 ms, DP=11) in posterior translation at the larger intensity (p=0.004), as shown in Figure 2.

**Figure 3:**
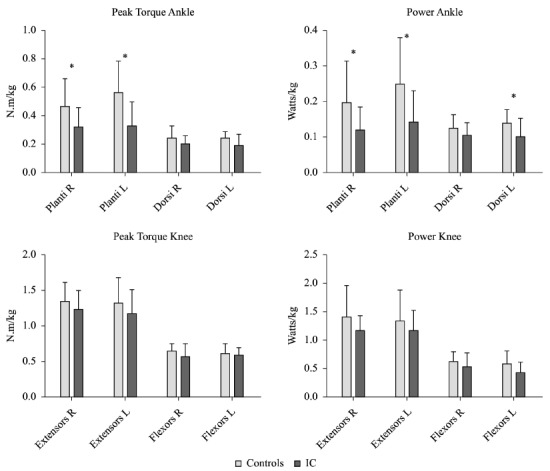
- Muscle performance. Peak torque and muscle power of dorsiflexors and plantar flexors in the ankle and the knee flexors and extensors of both limbs (right and left). * = p≤0.05.

The ICG showed decreased levels in muscle strength and power output. The results were statistically significant for the peak torque and muscle power in the right plantar flexors (*p* =0.036 and *p* =0.037) and left plantar flexors (*p* =0.008 and *p* =0.011), respectively, and muscle power of the left dorsiflexors (*p* =0.025). The extensors and flexors of the knee, in turn, showed no significant differences in peak torque or muscle power, but lower values were observed in the ICG (Figure 3).

## Discussion

The present study investigated postural control and muscular torque and power output in individuals with intermittent claudication, factors that may increase the risk of falls. The authors hypothesized that individuals with IC would have a greater deficit of balance and because of that, an increased risk of falls. However, the results of this study led the authors to partially reject the hypothesis. The IC participants showed dynamic postural control changes over the platform only in backward translation at the medium intensity perturbation. The ICG showed higher and faster shifts during this disruption and longer latency during the large translation.

The backward translation of the platform caused the body to oscillate forward due to the displacement of the center of mass anteriorly. To regain balance, the individual needs to reposition the body by shifting his/her center of mass back to the starting position. Research on the elderly^26^^,^ 27 have shown that this population has a greater displacement of COP during the platform translation. According to Daley and Spinks^28^, the largest amplitude displacement causes slower responses in the recovery of instability, increasing the likelihood of falls.

In order to restore balance after a sudden disturbance of the COP forward (caused by backward translation of the platform), contraction of the posterior muscles of the leg and trunk are necessary. The torque applied around the ankle joint during a disturbance has been described as the first action taken to restore postural control^29^. In addition, two studies have shown that individuals with a significant weakness in their lower limbs^6^^,^ 30 may show more body sway since they do not generate adequate stabilization torques at the ankles.

The results of this study are consistent with the literature, since people with IC showed decreased peak torque in the ankle plantar flexors. This finding may have contributed to the decline in postural control when balance was perturbed with the translation of the platform. The ability to generate higher torque in the ankle joint has been associated with the ability to reduce the COP excursion^29^.

Furthermore, participants with greater rates of generation of muscular power may present better balance performance by having a greater reactive ability to control their center of mass^31^. Muscle power and reaction time have been described as the main parameters for fall prevention^32^. In our study, participants with IC showed statistically significant reductions in muscle power of the right plantar flexors and left dorsiflexors.

It is believed that a decrease in power of the dorsiflexors of only one limb is related to the right-hand dominance of most individuals. Studies have shown that muscle strength has a positive relationship with gait limitations in individuals with POAD^33^^,^ 34. It is known that the dorsiflexors play a fundamental role in locomotion. Thus, a decreased walking speed and lower levels of physical activity could further reduce the overall performance: especially muscle strength^12^ and consequently, the power of the non-dominant limb. Nevertheless, it is possible that change in the muscle phenotype may have occurred in the non-dominant limb due to neuromuscular dysfunction, such as atrophy^35^. In this context, Regensteiner et al.^36^ showed that in individuals with POAD, there is a 31% reduction in peak torque of the dorsiflexors and a 43% reduction in the plantar flexors when compared to healthy individuals. Thus, it is believed that the lower percentage reduction in the peak torque of the dorsiflexors - compared to the plantar flexors - could also explain the change in muscle power only in the non-dominant leg.

Additionally, the subjects' latency time was higher in the ICG during posterior translation at a higher (large) intensity. The platform translations went from medium to large intensity. The authors believe that the lower displacement speed (10 cm/s) and the exposure time (300 ms) at the medium intensity were insufficient to promote different latency times between the groups. Longer latency times have been associated with peripheral neuropathy^37^. Studies with IC participants had shown the association of IC with peripheral nerve dysfunction^11^. The present results are in agreement with Mockford et al.^13^ who assessed 54 subjects with IC using the MCT. They showed an increase in the latency by 24% in IC participants compared to a control group.

Of the six conditions evaluated with the SOT - static equilibrium test - no statistically significant differences were observed in the amplitude and average velocity of the COP between groups. According to Horak et al.^37^, dynamic tests are better when differentiating more homogeneous populations than static tests which usually do not require restoring balance. In addition, it is clear that the elderly, particularly those who had fallen, had a higher dependence on a step strategy to maintain and restore balance, (i.e., using the dynamic activity of the lower limbs to protect against falls). Thus, it is understandable that the findings of this study showed no significant difference in the static balance tests.

There are few studies using PDC in individuals with IC. Mockford et al.^13^, using the SOT, found alterations in 41% of individuals with intermittent claudication compared to a healthy group. The sensory system with greater impairment was the vestibular (52%), followed by the somatosensory (22%) and the visual (17%). Horak et al.^37^ suggested that the information from the somatosensory, visual and vestibular systems is dynamically redistributed to maintain balance. This information should be integrated into the central nervous system (CNS) so that the motor system is able to produce proper muscle contraction. Often, these sensory stimuli are redundant. The abundance of information is important in situations where some of the systems are deficient, so that the remaining systems may compensate for such restrictions^37^.

In this study, through the evaluation of some of the risk factors for falls, such as decreased postural control and muscle weakness, it was observed that individuals with IC were more likely to fall than subjects with no claudication: even for IC participants without history of previous falls. These results are relevant for clinical practice, since a fall may have a great impact on the quality of life of individuals, leading to restriction of physical activity^1^ and increased hospitalizations^2^. Therefore, the development of rehabilitation strategies that include ways to minimize the potential for falls in this population is important.

It can be suggested that in addition to decreased strength and muscle power, a low level of physical activity in individuals with intermittent claudication (50% without regular physical activity) may be associated with balance disorders. A recent meta-analysis has shown that regular exercise significantly reduced the rate of falls in the elderly^38^.

Finally, some limitations of this study need to be observed. The cross-sectional design does not allow causality to be established. The large proportion of males in the sample can target our findings towards to the male population. Furthermore, the sensory evaluation was not performed to prove proprioceptive deficits in individuals with IC.

For Boucher et al.^39^, the sensory information, originating from cutaneous receptors in the plantar region in individuals with peripheral neuropathy, may influence postural control. However, this study showed no significant differences in postural control under conditions 1 and 2 of the SOT. Such conditions evaluated the ability of individuals to use the sensory inputs of the somatosensory system, mainly from the contact of the feet with the support surface to maintain balance. In addition, measurement of cutaneous sensitivity was not our goal in this study, since the Rutherford (gold standard)^40^ and ITB (sensitivity of 95% and specificity of 99% for the diagnosis of PAD) classifications were used to categorize the sample, similar to previous studies.

## Conclusion

IC is a condition that adversely affects the functional capacity of the individual, such as a decreased walking distance, changes in the level of muscle strength and power around the ankle joint and an impaired ability to regain balance after unexpected disruptions or perturbations. These results indicate that individuals with IC may become more susceptible to falls than individuals without IC.
